# A Parametric Study of the Effects of Critical Design Parameters on the Performance of Nanoscale Silicon Devices

**DOI:** 10.3390/nano10101987

**Published:** 2020-10-09

**Authors:** Faraz Kaiser Malik, Tariq Talha, Faisal Ahmed

**Affiliations:** Department of Mechanical Engineering, College of Electrical and Mechanical Engineering, National University of Sciences and Technology, Islamabad 44000, Pakistan; fmalik.me18ceme@student.nust.edu.pk (F.K.M.); faisal.ahmed@ceme.nust.edu.pk (F.A.)

**Keywords:** Joule heating, nanoscale silicon, Monte Carlo, 1D transport

## Abstract

The current electronics industry has used the aggressive miniaturization of solid-state devices to meet future technological demands. The downscaling of characteristic device dimensions into the sub-10 nm regime causes them to fall below the electron–phonon scattering length, thereby resulting in a transition from quasi-ballistic to ballistic carrier transport. In this study, a well-established Monte Carlo model is employed to systematically investigate the effects of various parameters such as applied voltage, channel length, electrode lengths, electrode doping and initial temperature on the performance of nanoscale silicon devices. Interestingly, from the obtained results, the short channel devices are found to exhibit smaller heat generation, with a 2 nm channel device having roughly two-thirds the heat generation rate observed in an 8 nm channel device, which is attributed to reduced carrier scattering in the ballistic transport regime. Furthermore, the drain contacts of the devices are identified as critical design areas to ensure safe and efficient performance. The heat generation rate is observed to increase linearly with an increase in the applied electric field strength but does not change significantly with an increase in the initial temperature, despite a marked reduction in the electric current flowing through the device.

## 1. Introduction

In a solid-state device, charge carriers interact with the lattice vibrations (phonons) of a material under an externally applied electric field. As a result, some of the applied electrical energy is converted into thermal energy; a phenomenon called Joule heating [1,2]. The Joule heating in electronic devices is a significant hindrance to their safe functionality and efficient performance. It is vital for most of the electronic components, such as integrated circuits, microprocessors and control systems, to maintain the low operating temperatures (below ~85 °C) to ensure durability, stable operation and prevention of thermal hazards [3,4].

The Joule heating effect becomes more challenging in modern electronics due to the recent developments in micro- and nano-scale technologies. The miniaturization of solid-state electronic devices has progressed at an exponential scale in line with Moore’s law, with transistor density doubling roughly every two years [5]. As a result, the 10 nm node has already been realized [6]. Simultaneously, Dennard scaling, which postulates that the transistor power density remains constant as dimensions are reduced, broke down around 2006, meaning that the modern electronic components are unable to operate within the same power envelope [7,8]. Therefore, the static power losses increase rapidly as a proportion of the overall supplied power with decreasing device dimensions and applied voltages. In addition to this, the static power losses are also a function of the device temperature, and thus directly depend upon the total dissipated power [9].

More importantly, the increased static power losses at smaller dimensions adversely affect the thermal conductivity of materials due to phonon confinement effects and the increased boundary scattering [1,10]. These enhanced scattering events increasingly randomize the momenta of phonons, raising the internal energy of the device, and consequently its operational temperature. These factors, along with enhanced chip density, have led to a drastic increase in the power density for high-performance processors. Furthermore, the increased thermal impedance in smaller devices leads to localized hotspot formation, which is a major concern for future circuit design. Therefore, reconsideration of cooling techniques and related favorable device structures with optimized design and performance parameters are necessary to prevent thermal runaway and breakdown.

For a conventional bulk electronic system, the classical Fourier’s law is employed to analyze the self-heating problems. For nanoscale electronics, wherein the physical size of the device is smaller than the phonon mean free path (200–300 nm for silicon at room temperature [11]), Fourier’s law is no longer applicable. At length scales comparable to or shorter than the phonon wavelength (1–2 nm for silicon at room temperature [12]), and/or at temperature values much lower than the Debye temperature, the quantum-mechanical behavior of phonons becomes important, which necessitates the use of atomistic approaches like the lattice dynamics equations or the ab initio method [12,13,14,15]. However, despite aggressive miniaturization, the dimensions (and temperatures) involved in modern electronic devices are not yet as small (low) to require quantum-mechanical modelling, and semi-classical methods such as the Boltzmann transport equation (BTE) suffice.

The BTE method incorporates the effects of external electric fields, electron–phonon interactions, phonon decay and various scattering mechanisms in its self-consistent formulation of phonon and electron transport [13]. The direct solution of the BTE is a cumbersome process and analytical solutions only exist for simple heat conduction problems [16]. Alternate approaches to quantify heat generation in nanoscale electronic devices include the drift-diffusion, hydrodynamic and Monte Carlo (MC) simulation methods, which are based on the numerical solution of the BTE. The drift-diffusion and hydrodynamic models are unable to identify the contribution of the individual phonon modes to heat generation [17,18,19]. Therefore, the determination of the energy-dependent scattering rates in mesoscopic devices with the desired level of accuracy becomes challenging. In contrast, the MC method, despite being stochastic in nature, provides detailed information about phonon generation and the exchange of energy between the charge carriers and the lattice [18]. This is of critical importance because different phonon modes with different group velocities exhibit varying contributions to heat generation and confinement in electronic devices.

MC simulations have been extensively used in literature to develop an understanding of how changes in device design, material and dimensions affect heat generation profiles. The ability to incorporate an accurate treatment of the various scattering mechanisms, and the lack of need to make assumptions regarding the carrier distribution in energy space [20] make the MC method a comprehensive approach for simulating charge transport in semiconductors. Pop et al. [21] developed an MC model that incorporated analytical descriptions for the electron band structure and the phonon dispersion relationship of silicon, while neglecting the impact ionization, thus reducing the computational time significantly, but making it suitable only for low-field applications. The same model was further extended to analyze the volumetric heat generation rate in ultra-short silicon devices and to draw comparisons with the drift-diffusion approach [22].

Electron–electron interactions were first incorporated in the MC simulations of nitride devices by Ashok et al. [23]. These interactions were found to cause the charge carriers to lose energy more rapidly near the drain contact of the device, compared to when they are not included in the scattering model. Harada et al. [24] applied the semi-classical MC method to silicon and monolayer graphene devices, and compared the differences in electron transport properties that arise due to the linear band dispersion of graphene. Shomali et al. [25] performed energy carrier transport simulations using the MC method to investigate the effects of boundary conditions on the temperature distribution profiles and localized hotspots in silicon nanodevices. Fang et al. [26] demonstrated the contribution of both acoustic and optical phonon emission to the energy loss in silicon and germanium devices through full-band MC simulations. Nghiem et al. [15] studied the temperature distribution and heat confinement resulting from ballistic transport in a nanoscale silicon sample heated through the top surface by a localized heat source.

Previous studies report that the electron–phonon mean free path lies between 5–10 nm for silicon under typical device operating conditions [22]. With the industry now aiming for the 5 nm lithography node, it is imperative that the behavior of silicon-based nanoscale electronic devices is studied to develop a fundamental understanding of the impact of increasingly ballistic carrier transport on device performance. Despite extensive MC-based studies of the performance characteristics of sub-micrometer nanoscale devices, detailed investigations of the variation of ballistic-regime device performance with operating conditions are not reported in the literature. This paper, therefore, studies the effects of several potentially critical parameters of interest (applied voltage, channel length, electrode lengths, electrode doping and initial temperature) on the performance parameters (volumetric heat generation rate, electron energy and electron drift velocity) of 1D silicon n^+^nn^+^ devices in the ballistic transport regime. The applied voltage and device dimensions both are expected to have a significant role in the determination of carrier energies and velocities through their influence on the electric field distribution in the devices. The electrode doping influences the number of available charge carriers present in the devices, and in turn, the electrical resistance of these devices. The initial lattice temperature is also expected to have a significant impact on carrier mobility and the rate of Joule heating in these devices.

## 2. Materials and Methods

**Figure 1:**
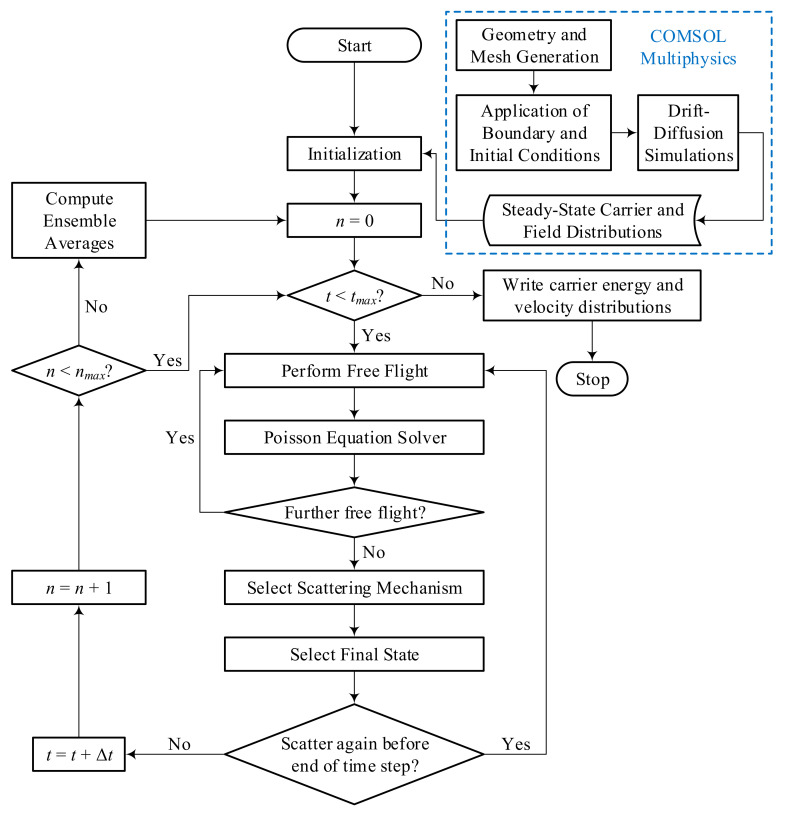
Flowchart of the ensemble Monte Carlo method used in this study. $t_{max}$ represents the total simulation time, $n_{max}$ represents the number of time steps that constitute a single sub-history and n represents the number of time steps that have passed since the last ensemble average computation.

The basics of the ensemble Monte Carlo method as applied to charge transport in semiconductors have been described in detail by Jacoboni and Lugli [27]. Figure 1 presents a flowchart of the basic steps of the MC model employed in the current study—the simulations have been performed through remote access of the supercomputer at the Research Center for Modelling and Simulation, National University of Sciences and Technology, Islamabad, Pakistan. Several thousand super-particles, each in turn representing billions of real electrons, are used in the transient MC simulations to represent the mobile charge inside the semiconductor. Information about the device geometry, applied electric field and mesh setup, as well as the initial conditions for carrier concentration and doping profiles, are obtained from the output of preliminary simulations performed on a commercial device simulator based on the drift-diffusion model (COMSOL Multiphysics [28], COMSOL Inc., Stockholm, Sweden). This eliminates the potential convergence issues associated with randomly initialized distributions and thereby enables faster MC computations [29].

The MC model and related program code ‘MONET’ developed by Pop et al. [21,30], which uses an analytical non-parabolic band approximation for electron energy has been employed for this investigation. This approximation, which significantly reduces the computational time in comparison to full-band methods, is justified for sub-bandgap applied voltages and low energy studies, since impact ionization is insignificant at these conditions. For the non-parabolic conduction band with ellipsoidal equi-energetic surfaces, the relationship between the electron energy, ε, and the wavevectors, $k_{i}$, can be expressed using the many-valley model as [27]:

(1) $$\varepsilon \left(1+\alpha \varepsilon \right)=\frac{\hbar^{2}}{2}\left(\frac{k_{x}^{2}}{m_{x}}+\frac{k_{y}^{2}}{m_{y}}+\frac{k_{z}^{2}}{m_{z}}\right),$$

where $m_{i}$ is the component of the effective electron mass tensor along the ith direction and ℏ is the reduced Planck’s constant. The temperature dependence of the non-parabolicity parameter α for silicon was incorporated as [22]:

(2) $$\alpha =0.5\frac{E_{g_{0}}}{E_{g}\left(T\right)},$$

where $E_{g_{0}}$ is the bandgap energy at room temperature, and $E_{g}\left(T\right)$ is the temperature-dependent bandgap, which, for silicon, can be expressed in terms of the absolute temperature T as [31]:

(3) $$E_{g}\left(T\right)=1.1756-\left(8.8131\times 10^{-5}\right)T-\left(2.6814\times 10^{-7}\right)T^{2}$$

Therefore, at room temperature, α=0.5 eV^−1^, while using α=0 results in the original parabolic model of Canali et al. [32].

In the MC simulation, the super-particles are initialized with randomly oriented momenta in numbers proportionate to the mobile charge carrier distribution imported from COMSOL. The charge carriers are then subjected to free flight under the influence of an external electrical field for a certain time interval determined stochastically based on pre-defined scattering event probabilities. Upon undergoing scattering, a new electron state (ε, k) is randomly chosen as the initial state for the next free flight interval, while accounting for energy and momentum conservation using a rejection algorithm [21,33]. The process then repeats iteratively until the desired precision is achieved. A fictitious self-scattering event is also introduced, which allows the charge carrier to continue its free-flight unimpeded by assigning it the same energy state after the event as before. In this way, the total scattering probability (through all mechanisms, including self-scattering) remains constant and independent of carrier energy. For the non-parabolic band model employed here, the electron velocity v associated with a particular state is determined as [27]:

(4) $$v\left(k\right)=\frac{1}{\hbar }\frac{\partial \varepsilon }{\partial k}=\frac{\hbar k}{m\left(1+2\alpha \varepsilon \right)},$$

where m is the effective mass. Moreover, the total simulation time $t_{max}$ is divided into multiple sub-histories over which intermediate ensemble averages are calculated. In Figure 1, $n_{max}$ is the number of time steps over which the intermediate ensemble averages are to be computed (a single sub-history), while n represents the number of time steps that have been simulated since the last ensemble averages were computed. An initial portion of the simulation is not included in the computation of final ensemble averages to exclude the transient effects of the initial condition selection.

Furthermore, the model treats all phonon scattering events inelastically to accurately capture the energy dissipation information for the heat generation calculations. The contribution of each phonon dispersion branch is separately accounted for, with the following analytical isotropic approximation applied:

(5) $$\omega_{q}=\omega_{0}+v_{s}q+cq^{2},$$

where $\omega_{q}$ is the phonon frequency, q is the phonon wave vector and the values of the parameters $\omega_{o}$, $\nu_{s}$ and c, which have units of s^−1^, cm s^−1^, and cm^2^ s^−1^ respectively, are chosen separately as below for each of the four phonon modes to replicate the experimentally determined dispersion [21,30]:

(6) $$\left(\omega_{0},v_{s},\;c\right)\left\{\begin{array}{cccc} (0, & 9.01\times 10^{5}, & -2.00\times 10^{-3}) & \mathrm{Longitudinal}\;\mathrm{Acoustic}\\ (0, & 5.23\times 10^{5}, & -2.26\times 10^{-3}) & \mathrm{Transverse}\;\mathrm{Acoustic}\\ (9.88\times 10^{13}, & 0, & -1.60\times 10^{-3}) & \mathrm{Longitudinal}\;\mathrm{Optical}\\ (10.20\times 10^{13}, & -2.57\times 10^{5}, & 1.11\times 10^{-3}) & \mathrm{Transverse}\;\mathrm{Optical} \end{array}\right.$$

Ionized impurity scattering, which significantly affects the carrier mobility at high doping concentrations, is incorporated using the model developed by Kosina [34], also employed by Pop et al. [22]. The MC model computes the volumetric heat generation rate at steady state as the sum of the energies $\hbar \omega_{q}$ of all phonons emitted minus the energies of all phonons absorbed per unit volume over a certain simulation time $t_{sim}$ as follows:

(7) $$Q^{\prime \prime \prime }=\frac{1}{t_{sim}}\sum \left(\hbar \omega_{emitted}-\hbar \omega_{absorbed}\right).$$

The Poisson equation is also solved at regular intervals during free-flight to update the electric field self-consistently with the motion of the charge carriers. The super-particles are treated as individual charge carriers during free flight, but as clouds of charges for the Poisson equation solution. The updated charge distribution obtained through the solution of the equation is mapped to the mesh grid using the cloud-in-cell method, which performs a simple weighted linear interpolation of the charge of the cloud onto the adjacent grid nodes. This approach allows for realistic and more accurate simulations in comparison to models that maintain a fixed electric field regardless of charge carrier motion [27,29].

**Figure 2:**
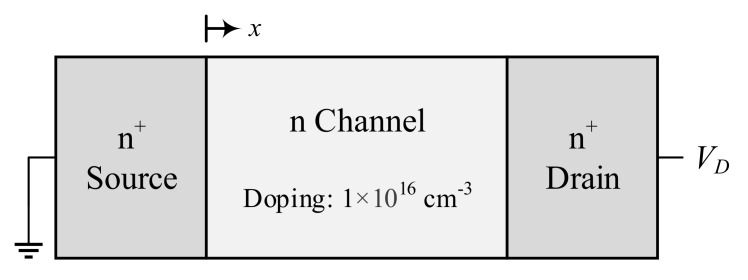
Schematic of the n^+^nn^+^ device investigated in this study. The source and drain lengths are varied from 20 nm to 150 nm, while the channel length is varied from 2 nm to 120 nm in this study.

Figure 2 shows a schematic of the 1D n^+^nn^+^ silicon device investigated in the present work, which is a simplified model of the cross-section of a MOSFET channel. This model has an energy band diagram similar to that of a MOSFET channel, with an injection barrier and a highly peaked lateral electric field, and includes impurity scattering and velocity overshoot, but the multi-dimensional potential gradients and confinement effects encountered in full device simulations are not present. This realistic 1D representation has thus been frequently used in the literature [20,22,23,24] to study the transport phenomena in electronic devices in the primary direction of carrier transport—which fundamentally determine the device performance—in isolation from the complicating factors that arise in a full device simulation together with an increase in the computation time and cost. Moreover, while the MONET program employed in this study is able to simulate carrier transport in two-dimensional geometries, it does not incorporate a Poisson equation solver for the 2D mode. This means that the electric field distribution cannot be updated self-consistently with the motion of charge carriers, rendering the program unable to provide quantitative results and greatly reducing the advantage in accuracy offered by the MC method over the much faster drift-diffusion method for 2D simulations [35].

The n-type channel of the 1D device shown in Figure 2 has homogeneous mild doping of 1 × 10^16^ cm^−3^ (almost intrinsic doping), while the channel length, electrode lengths, electrode doping and applied potential difference are varied systematically for this study. The potential difference across the boundaries is kept constant according to the potential profile obtained from a preliminary drift-diffusion simulation. A periodic boundary condition is applied to the device contacts to ensure current continuity, such that particles escaping from one contact are reintroduced at the other contact with thermal energy and inwards momentum p, calculated as:

(8) $$p=\sqrt{-2m_{x}k_{B}T\ln\left(r\right)}$$

where $k_{B}$ is the Boltzmann constant and r is a random number uniformly distributed between 0 and 1. A uniform grid is used to simplify the charge assignment process. The effects of grid resolution and the time step size were assessed in a preliminary study and the values of these parameters were carefully chosen to be Δx=0.25 nm and Δt=0.2 fs from the preliminary study to ascertain the accuracy of the simulations. Furthermore, after analyzing the convergence of the numerical results of this preliminary study, a total simulation time $t_{max}=100$ ps was chosen, with the number of time steps constituting a single sub-history taken to be $n_{max}=200$. The start of the channel is taken as the reference point of the x-coordinate, unless stated otherwise.

## 3. Results and Discussion

### 3.1. Validation

**Figure 3:**
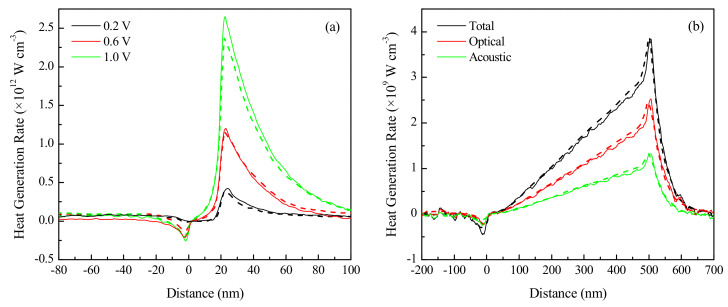
Volumetric heat generation rates at 300 K over the lengths of (**a**) 20 nm and (**b**) 500 nm channel n^+^nn^+^ devices. Solid lines are the results of this work, and dashed lines are the Monte Carlo (MC) results obtained from Pop et al. [22].

Prior to solving the problem of interest, preliminary simulations were performed to validate the performance characteristics of 1D quasi-ballistic n^+^nn^+^ devices, a schematic of which is shown in Figure 2. This comparison of results against published literature helps establish a level of confidence in the methodology adopted in the present study. Figure 3a compares our computed results with the published data sets of the heat generation profiles at three different applied potentials for a 20 nm long channel n^+^nn^+^ device with 10^20^ cm^−3^ electrode doping density.

As mentioned earlier, the employed methodology accounts for the heat generated through the optical and acoustic phonons separately. Therefore, the validation for the contribution of both types of phonons has also been performed. Figure 3b presents the results for the volumetric heat generation along the entire length of an n^+^nn^+^ device with a 500 nm long channel, wherein the device is subjected to a 2.5 V drain voltage ($V_{D}$) with an electrode doping density of 10^18^ cm^−3^. The obtained trends in Figure 3 are in good agreement with the published results of Pop et al. [22]. A maximum error of 11.8% is observed in Figure 3a at 1.0 V (close to the non-parabolic band approximation limit), while a maximum error of less than 6% is observed in the comparisons shown in Figure 3b. This deviation is within reasonable expectations due to the stochastic nature of the MC method.

### 3.2. Effect of Voltage

**Figure 4:**
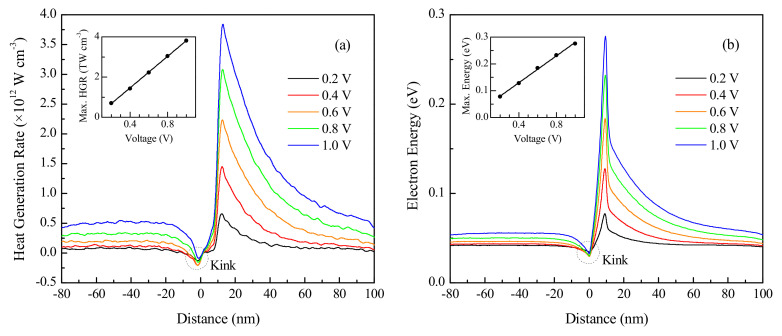
Effect of voltage on (**a**) heat generation and (**b**) electron energy over a 10 nm long channel n^+^nn^+^ silicon device.

It is observed from Figure 3a that changing the applied $V_{D}$ has a pronounced effect on the volumetric heat generation rate in the silicon device. Therefore, additional simulations were performed on the device by changing the applied potential difference. A 10 nm long silicon channel device with 100 nm long electrodes doped to 10^20^ cm^−3^ is used for this investigation. Since a non-parabolic band approximation is used, and the role of impact ionization is neglected in the employed methodology, only $V_{D}$ values up to 1.0 V are investigated for the smaller channel. Impact ionization, which becomes more significant at stronger field strengths, is a carrier multiplication process that increases the number of mobile charge carriers and must be modeled separately in high-energy applications due to the resulting reduction in carrier mean free path [36]. At high applied voltages, the lack of incorporation of impact ionization, therefore, causes the non-parabolic band approximation to incorrectly compute a higher total conduction band density of states [21], which in turn affects the carrier scattering rates. Nevertheless, the model is able to accurately simulate carrier transport for the range of operating conditions expected to be encountered in most low-voltage nanoelectronics. The results presented in Figure 4 are for an initial lattice temperature of 300 K.

Figure 4a,b presents the variations of the volumetric heat generation profile and the electron energy distribution with the applied potential difference, respectively. The maximum heat generation rate and the maximum electron energy are both found to increase linearly with the applied voltage, as evident from the corresponding insets of Figure 4a,b. Interestingly, this linear correlation is consistent with the results reported by Pop et al. [22] for a longer channel silicon device, which indicates that the effect of voltage on device performance does not break trend upon device miniaturization into the ballistic transport regime, unlike the other parameters investigated in this study. A higher potential difference across the device terminals causes the charge carriers to gain more energy as they traverse through the channel. The highly doped drain region contains cold electrons that do not contribute to the net heat generation rate. Hot electrons injected from the channel into the drain release their energy in discrete quantities through phonon emission over multiple scattering lengths. This causes the heat generation region to extend deep into the drain, contrary to being confined to the proximity of the channel. Furthermore, as indicated in Figure 4a, a small kink representing a region of negative heat generation, which is accompanied by a reduction in the electron energy at the same location in Figure 4b, is observed at the onset of the channel. This is due to the fact that the electrons extract heat from the lattice via net phonon absorption to surmount the interfacial potential barrier formed between the highly doped source and mildly doped channel region [18].

### 3.3. Effect of Channel Length

By comparing Figure 3a and Figure 4a, a significant increase in the heat generation rate for the corresponding voltages is observed as a result of the channel length being decreased from 20 nm to 10 nm. The effect of changing the channel length is studied in more detail by simulating devices with varying channel lengths between 2 nm and 120 nm and a constant electrode doping of 10^20^ cm^−3^. The devices are subjected to an applied $V_{D}$ of 0.8 V and an initial temperature of 300 K. The minimum investigated channel length is limited by the scale constraints on the Boltzmann equation, which serves as the basis for the MC method [12].

**Figure 5:**
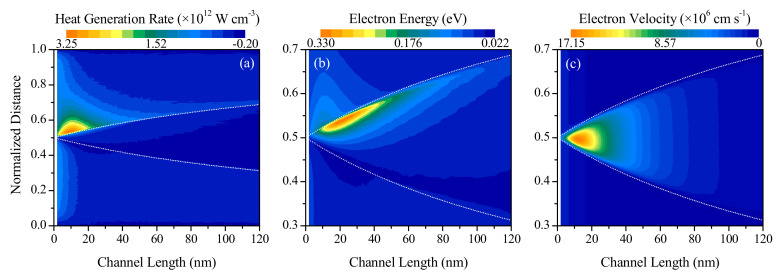
Variation of (**a**) heat generation rate, (**b**) electron energy, and (**c**) electron velocity as a function of the channel length of an n^+^nn^+^ silicon device. The lower and upper white dotted lines correspond to the start and end of the channel, respectively.

Figure 5 illustrates the effects of changing the channel length on the distributions of volumetric heat generation rate, electron energy and electron velocity. The reference of the x-coordinate is taken at the start of the device in this figure. Note that a normalized distance xxtotal of 0.5 corresponds to the center of the device, which coincides with the center of the channel since the source and drain have equal lengths of 100 nm. From Figure 5a,b, it is observed that the maximum heat generation and maximum electron energy for any given channel length occur along the channel-drain contact (consistent with the results shown in Figure 4), since the electrons gain most of their energy at this contact due to the abrupt peak in the electric field distribution here, and then proceed to dissipate this energy to the lattice atoms in the neighborhood. Meanwhile, the electron velocity, shown in Figure 5c, is found to be maximum in the intrinsically doped channel region, with a slightly higher velocity observed near the source end of the channel than at the drain end due to the injection of low-energy electrons from the heavily-doped drain.

**Figure 6:**
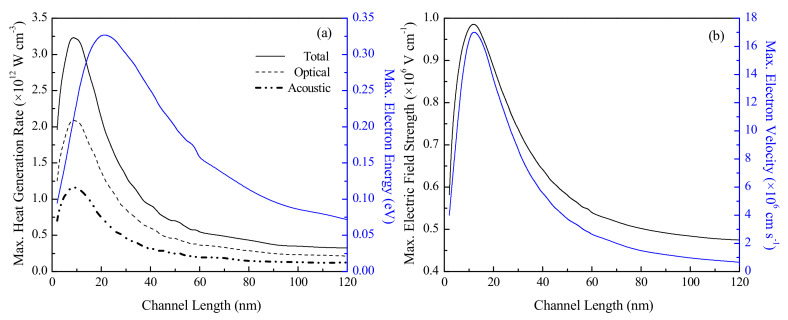
Variation of the maximum (**a**) heat generation rate and electron energy and (**b**) electric field strength and electron drift velocity in an n^+^nn^+^ device with channel length at 300 K. In (**a**), the separate contributions of the optical and acoustic phonon modes to the total heat generation rate are also shown.

Figure 6 presents the variation of the parameter maxima with the device channel length. The contribution of the optical phonon mode to the heat generation rate is observed to be almost twice as much as that of the acoustic mode at any given channel length. This is explained by the difference in phonon group velocities [9], with the slower optical phonons contributing little to thermal conductivity and dissipating a greater portion of energy to the lattice locally in comparison to their acoustic counterparts.

The maximum volumetric heat generation rate, maximum electron velocity and maximum electron energy show peaks at 8 nm, 12 nm and 20 nm respectively. Considering the range of maximum electron velocities observed here, and assuming an average scattering time based on values reported in the literature, the inelastic electron-phonon scattering length under the simulated conditions is found to be in between 5 and 10 nm [22]. For channel lengths shorter than this scattering length, which is the length scale of primary interest in this study, the number of scattering events greatly decreases as ballistic transport starts to dominate. This causes the electrons to lose less energy to the lattice, and in turn results in reduced heat generation—the maximum volumetric heat generation rate in a 2 nm channel device is found to be ~61% of that in an 8 nm channel device. These findings also verify the observations of Rowlette et al. [37], that the prevalence of near-ballistic conditions within the channel are the likely reason behind the indications of their results for a 20 nm channel n^+^nn^+^ device. On the other hand, the reduction in both dynamic and leakage power with increasing channel length above 10 nm is a well-explained phenomenon [9,38]. For the same applied $V_{D}$, a longer channel length implies a weakened driving electric field, which subsequently results in a decrease in the current flowing through the device. The reduced current corresponds to a reduced rate of power dissipation, which causes the maximum volumetric heat generation rate in a 40 nm channel device fall to ~28.4% of that observed in an 8 nm channel device, and explains the observed peak in the maximum heat generation rate.

The maximum electric field across the channel region for the same applied potential difference is found to decrease with a shortening channel below 12 nm, which directly affects the maximum electron velocity, and in turn, electric current. This is attributed to the direct source–drain tunneling that results from an increasingly ballistic nature of transport as device dimensions are downscaled, which causes an increased potential drop across the electrodes and can potentially culminate in thermal runaway and breakdown. The tunneling phenomenon gives rise to a large leakage current, which ultimately causes an increase in power dissipation [39]. The reduction in heat generation rate for channel lengths below 8 nm does not, therefore, guarantee an improvement in device performance.

### 3.4. Effect of Electrode Lengths

**Figure 7:**
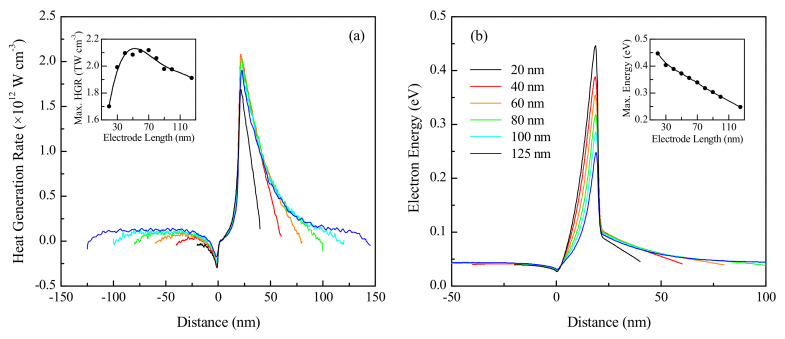
Effect of electrode length on the (**a**) heat generation rate and (**b**) electron energy over the length of a 20 nm channel device at 300 K (legends identical).

The effect of changing the electrode length on the device performance in the ballistic regime was investigated by performing simulations on a 20 nm channel device subjected to an applied $V_{D}$ of 0.8 V, with an electrode doping of 10^20^ cm^−3^. Since it was anticipated that the primary influence of a reduction in the electrode lengths would be to hamper the ability of the energized charge carriers injected into the drain to completely dissipate their excess energy and return to thermal equilibrium, the chosen channel length (20 nm) corresponds to the length at which the electron energy was observed to be maximal in Figure 6. The heat generation rate and electron energy profiles over the length of the devices are presented in Figure 7.

As shown in Figure 7a,b respectively, the heat generation rate and electron energy both are higher for the longer source and drain regions. Elongating the electrodes results in a slightly stronger effective electric field across them for the same applied potential—causing a small increase in electron energy—while also increasing the number of scattering events, therefore resulting in increased heat dissipation. For the shorter devices (20 to 40 nm electrode length), the heat generation trends indicate that the lattice is unable to entirely dissipate the energy imparted to it by the charge carriers in the drain region. Since the energy is carried in discrete quantities by phonons, which can only lose energy through the scattering events, it is important that the drain length and device contacts be adequately designed to ensure safe and efficient operation. These findings are consistent with those of previously reported studies [22,40] in which it has been noted that heat generation in mesoscopic devices is expected to be significant along the contacts instead of the active device region.

With increasing electrode length, the maximum heat generation rate in the channel, shown in the inset of Figure 7a, is found to increase significantly for the shorter electrode devices, followed by a relatively gradual decrease. The inset of Figure 7b indicates decreasing maximum electron energy for increasing electrode lengths, with the trend being linear for electrodes longer than 30 nm. Longer electrodes result in a weaker electric field across the channel region for the same applied $V_{D}$, resulting in the charge carriers undergoing less acceleration across the intrinsically doped channel. This subsequently causes a reduction in the maximum electron energy and maximum heat generation rate, both of which are observed at the drain end of the channel. Despite the reduced maximum heat generation at smaller electrode lengths, the charge carriers do not come into thermal equilibrium with the semiconductor lattice prior to leaving the device, and the excess energy is dissipated at the critical device junctions, which can result in inefficient operation and thermal breakdown. These findings, therefore, emphasize the need for adequately long electrodes to ensure that thermal equilibrium is achieved within the drain region.

### 3.5. Effect of Temperature

**Figure 8:**
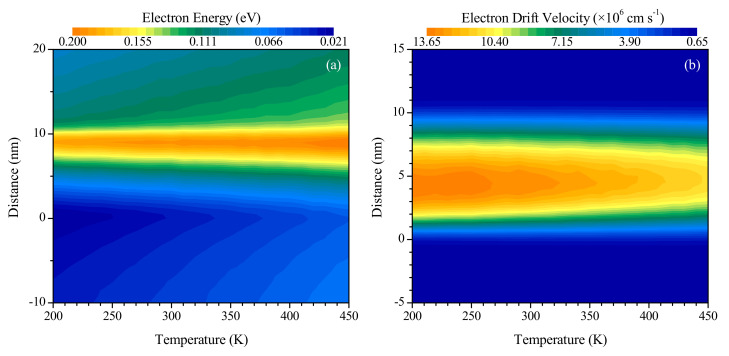
Variation of the distributions of (**a**) electron energy and (**b**) electron drift velocity over a section of the total device length with initial temperature.

**Figure 9:**
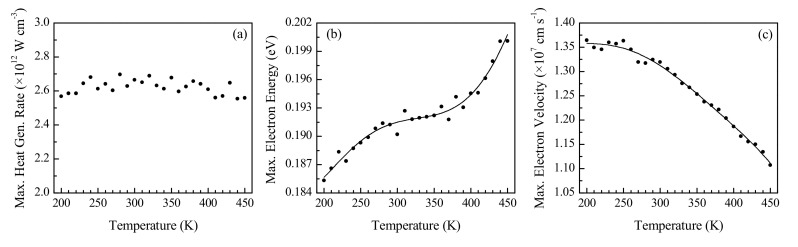
Effect of initial temperature on the maximum (**a**) volumetric heat generation rate, (**b**) electron energy and (**c**) electron drift velocity in a 10 nm channel n^+^nn^+^ device.

The effect of the initial lattice temperature on the device performance was investigated by performing simulations on a 10 nm long channel silicon device with 150 nm long source and drain regions doped to 10^20^ cm^−3^ at $V_{D}=$ 0.8 V. Since the lattice temperature is known to influence the carrier drift velocity and mobility in bulk silicon [21,31,32], the device channel length is chosen to be 10 nm for this investigation since this value is close to the length (12 nm) at which the highest maximum electron drift velocity was observed in Figure 6b, while also being close to the length (8 nm) at which the highest maximum heat generation rate was observed in Figure 6a. Figure 8 illustrates the effects of temperature on the distributions of electron energy and drift velocity over a section of the device length, while Figure 9 illustrates the variation with temperature of parameter maxima evaluated over the entire device length.

From Figure 8a, the increase in average electron energy with temperature over the length of the device is evident, while Figure 8b indicates a decrease in the average electron drift velocity as the initial lattice temperature is raised. Please note that the figures show the distributions in the vicinity of the channel (channel ranges from 0≤x≤10 nm), which is the region of significant changes in the distributions due to the presence of the peaked electric field. It is also observed that, consistent with the previous results of Figure 5, the maximum electron energy occurs near the drain end of the channel, while the maximum drift velocity occurs closer to the source end of the channel for the range of temperatures investigated in the present study.

Figure 9a indicates that no significant correlation between temperature and the maximum heat generation rate in the device exists. This is attributed to the effects of increasing temperature on the maximum electron energy (seen to increase in Figure 9b) and the maximum electron drift velocity (seen to decrease in Figure 9c). The electron energy increases since the temperature is directly linked to the internal energy of the lattice, with a higher temperature involving the promotion of electrons to higher energy bands [41]. The reduction in the rate of increase of the maximum electron energy at moderate temperatures, as observed in Figure 9b, may be attributed to the band structure of silicon limiting the promotion of the highest energy electrons into higher energy valleys at these temperatures, since the average electron energy, both over the full length of the device and over the length of the channel, as determined using the results presented in Figure 8a, increases linearly throughout the range of temperatures investigated. Higher electron energy leads to an increase in heat generation, since a larger amount of energy can be dissipated to the lattice through inelastic electron-phonon scattering events. The maximum drift velocity, on the other hand, decreases due to the increased vibrational motion of the lattice atoms, which impedes the motion of the charge carriers and results in velocity saturation [21,32]. This, in turn, reduces the current flowing through the device for the same applied potential difference. The overall effect of a higher initial temperature is thus a decreased rate of flow of higher energy carriers, culminating in a negligible net effect on heat generation rate for the range of temperatures considered. Because the average electron energy continues to increase at a constant rate, the slight reduction in the rate of increase of the maximum electron energy at moderate temperatures is not observed to have a significant impact on the maximum heat generation rate.

It is interesting to note that the variation of the maximum drift velocity is phenomenologically consistent with the effect of temperature on ZnO diodes with a 250 nm long channel as reported by Adeleh and Reza [20]. However, in their investigations, the maximum electron energy was found to decrease slightly with an increase in temperature, unlike the present findings. This was despite the average electron energy being higher at higher temperatures, similar to the present results seen in Figure 8a. This difference is best explained by the significantly different band structures and bandgap energies (3.4 eV for ZnO [20] compared to 1.1 eV for silicon [21]) of the two materials.

### 3.6. Effect of Electrode Doping Density

**Figure 10:**
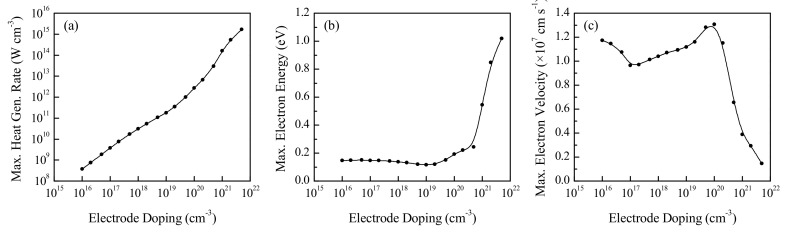
Effect of electrode doping on the maximum (**a**) volumetric heat generation rate, (**b**) electron energy and (**c**) electron drift velocity in a 10 nm channel n^+^nn^+^ device.

Furthermore, the effect of electrode doping density on device performance is also investigated. For this study, the 10 nm long channel silicon devices with 150 nm long source and drain regions doped to varying levels, subject to an applied $V_{D}$ of 0.8 V are considered. The electrode doping levels for the simulations range from 10^16^ cm^−3^ (equal to the channel doping) to 5 × 10^21^ cm^−3^. The choice of channel length, which lies in a potentially critical range as indicated by the results of Figure 6, aims to best capture the effects of changing the doping density, which is known to affect the electron drift mobility in bulk silicon [27,30]. Figure 10 shows the effects of the electrode doping density on the different parameters. Please note that these plots are shown on log–log and semi–log scales.

Figure 10a indicates that the maximum volumetric heat generation rate increases continuously with increasing electrode doping, while Figure 10b indicates an initial gradual decrease in electron energy at low electrode doping levels, followed by a gradual increase that becomes sharper as the doping level becomes degenerate. At an electrode doping level equal to the channel doping (10^16^ cm^−3^), the potential barrier between the channel and electrode regions is nonexistent, with the device essentially being one continuous semiconducting element. As the electrodes become more heavily doped, the potential barrier increases until the semiconductor becomes degenerate and behaves more like a metal. The carrier mobility in highly doped silicon is known to decrease with increasing doping levels due to enhanced carrier scattering [42], which explains the reduction in maximum drift velocity at high doping levels seen in Figure 10c. At low doping levels, the drift velocity is high, as there is no potential barrier to overcome, and the velocity decreases as the barrier develops. The drift velocity then increases again at moderate doping levels, since the carriers that overcome the barrier at the source–channel interface undergo a greater acceleration due to a sharper reduction in the conduction band energy level in the channel.

## 4. Conclusions

In this study, the effects of changing the applied electric field, channel length, electrode lengths, electrode doping and initial lattice temperature on the heat generation, electron energy and electron velocity distributions in nanoscale 1D n^+^nn^+^ silicon devices have been investigated through an established ensemble Monte Carlo simulation model. Given the continued focus on device miniaturization, the results of this study are of particular significance to the development of modern electronic devices with a characteristic length scale lying in the ballistic transport regime.

The performance parameters are found to vary linearly with the applied voltage, whereas mutually offset maxima have been observed by varying the channel length due to transition into the ballistic regime. These maxima indicate a significant departure from the performance trends known for longer devices operating in the quasi-ballistic transport regime. Furthermore, results for the impact of the electrode lengths are found to be primarily dependent upon the variation of the potential difference across the three device regions with electrode length, and emphasize the necessity of careful drain and contact design in ultra-short devices, since most of the heat dissipation occurs in the drain region. Given the spatial constraints that are compounded by miniaturization, the incorporation of a heat sink in the vicinity of the drain should thus be of preferential importance.

Moreover, the results indicate that it is essential to consider the trade-off between a reduction in the maximum heat generation rate and a simultaneous reduction in the electrical and thermal conductivities—which leads to inefficient operation and may even cause thermal runaway—as device dimensions are scaled-down. An increase in initial lattice temperature does not have a significant effect on the heat generation rate due to the counteracting contributions of the effects on carrier energy and velocity. The heat generation rate is found, however, to increase with an increase in electrode doping.
